# Supplementary material for: Biological Pathway-Derived TMB Robustly Predicts the Outcome of Immune Checkpoint Blockade Therapy
Source: Cells. 2022 Sep 8;11(18):2802. doi: 10.3390/cells11182802 (PMC9496944; doi:10.3390/cells11182802)
Supplement: Supplementary file 1 [file cells-11-02802-s001.zip › cells-1680519-supplementary.pdf]

cells MDPI

Dear Editors and Reviewers,

Thanks for your review of our manuscript entitled “Biological pathway derived TMB robustly predicts the outcome of immune checkpoint blockade therapy” (Manuscript ID: cells-1680519). We appreciate the concerns and suggestions provided by you and the reviewers, and have revised our manuscript accordingly.

Comments

1. For primary melanoma, the location on the skin has to be indicated. I attach an example. It can be less categories, but it is helpful to sort more specific descriptions from the patient journal into such widely accepted categories to facilitate comparision with other studies.

2. If the biopsy was taken from a melanoma metastasis, it should be explicitly named "metastasis". The anatomical position is in that case not so important, but can be named.

3. If there is no information about the location, it is OK to write "no information". Do not write "melanoma tumor biopsies".

4. Please recheck: "subcutaneousalp, subcutaneous"; "metastasos", "parietal metastasis", "abdonimal nodule"

5. For the overview, all metastatic melanoma are one category. Melanoma without information on location is another category. As it appears that the large majority of your samples were taken from metastases, it might not be necessary in the overview to subdivide the primary melanoma according to location, since the percentages would be rather small. So primary melanoma could be one category for the overview (But it is still important to keep the detailed information in the Table S1, of course!)

6. It is sufficient to give the percentage of male biological gender, as the percentage of the female gender follows from that.

7. Write "+/−" instead of "standard deviation" in the result text.

Response: Thanks for the valuable suggestions. We have carefully checked and revised the clinical information presented in Table S1 (marked in red) and revised the corresponding description in the method section of the manuscript as follows:

"For melanoma, age of the patients ranged from 18 to 90 (average: 60±14), and male patients accounted for 67.7%. Majority tumor samples were taken from melanoma metastasis (90%), and the left were taken from the primary melanoma. All samples were taken at stage IV. For NSCLC, age of the patients ranged from 41 to 85 (average: 63±9), and male patients accounted for 51.3%. All samples were taken from the lung tumor at stage IV."

We appreciate the time, expertise, and remarks of all reviewers and editors very much. Upon review of our revised manuscript, we hope that you will find it acceptable for publication on ***Cells***.

Sincerely,

An-Yuan Guo

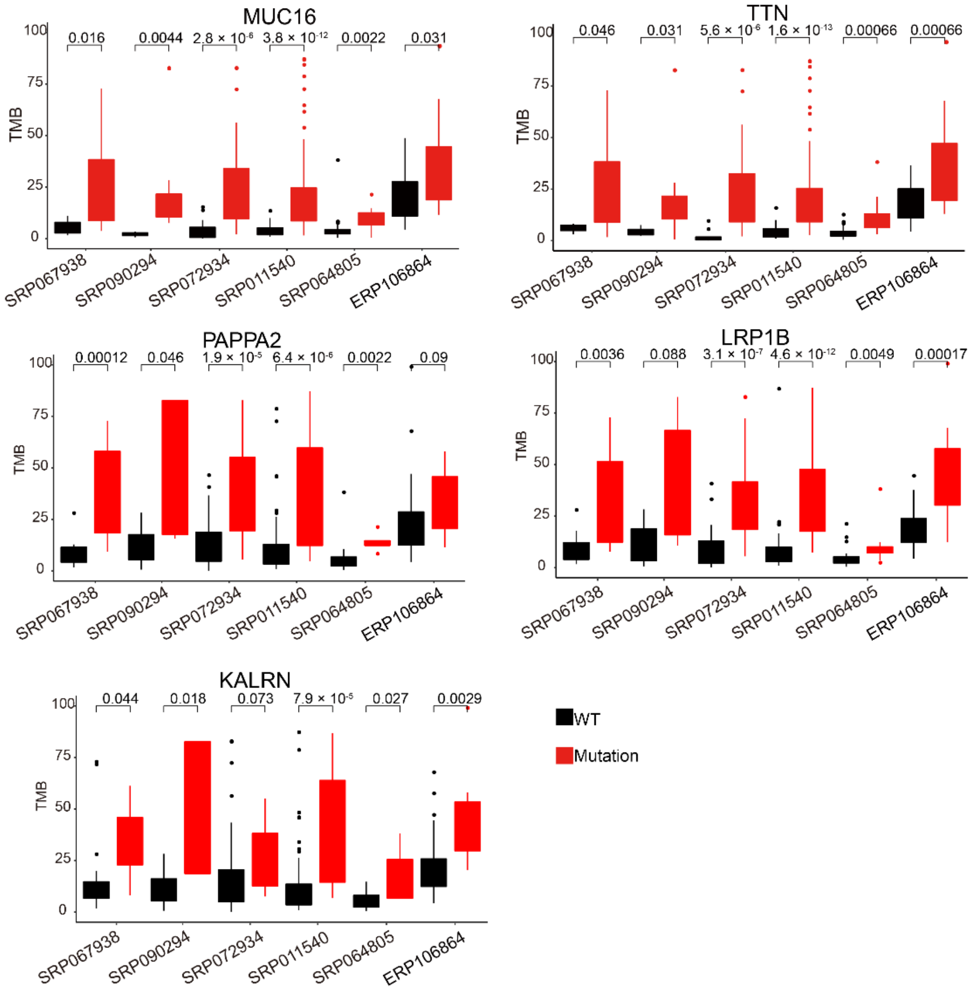

Figure S1. Genes that its mutation was significantly correlated with high TMB in all six datasets. TMB was significantly higher in patients with the mutant gene (red color) than in those with wild-type gene (black color). p-value is estimated by Mann-Whitney U test.

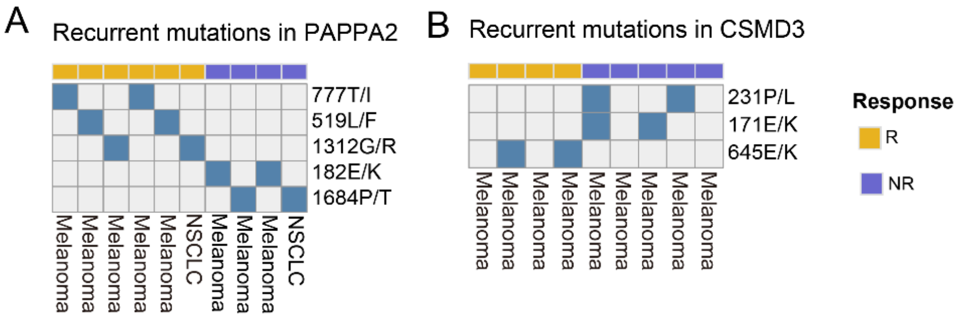

Figure S2. Some mutant loci in PAPPA2 (**A**) and CSMD3 (**B**) occur only in response or non-response samples.

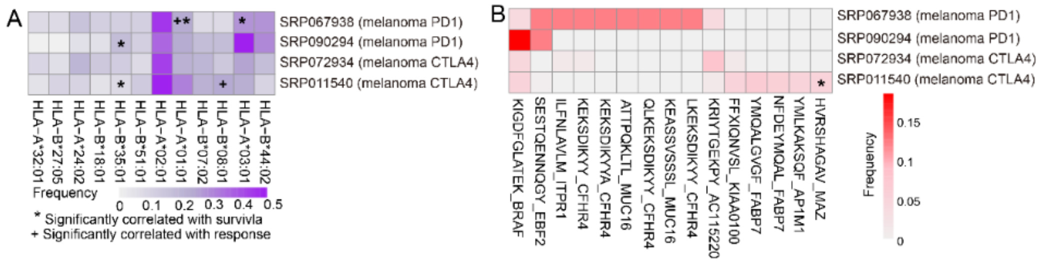

Figure S3. Recurrent HLAs (**A**) and neoantigens (**B**) in four melanoma datasets. "*" means HLA subtypes were significantly correlated with patient survival (log-rank test p-value < 0.05), and "+" means HLA subtypes were significantly associated with patient response (fisher's exact test p value < 0.05).

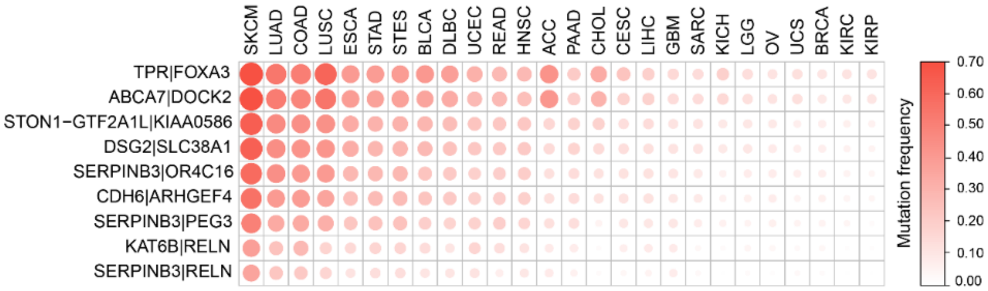

Figure S4. Frequency of survival related gene pairs in TCGA cancer samples.

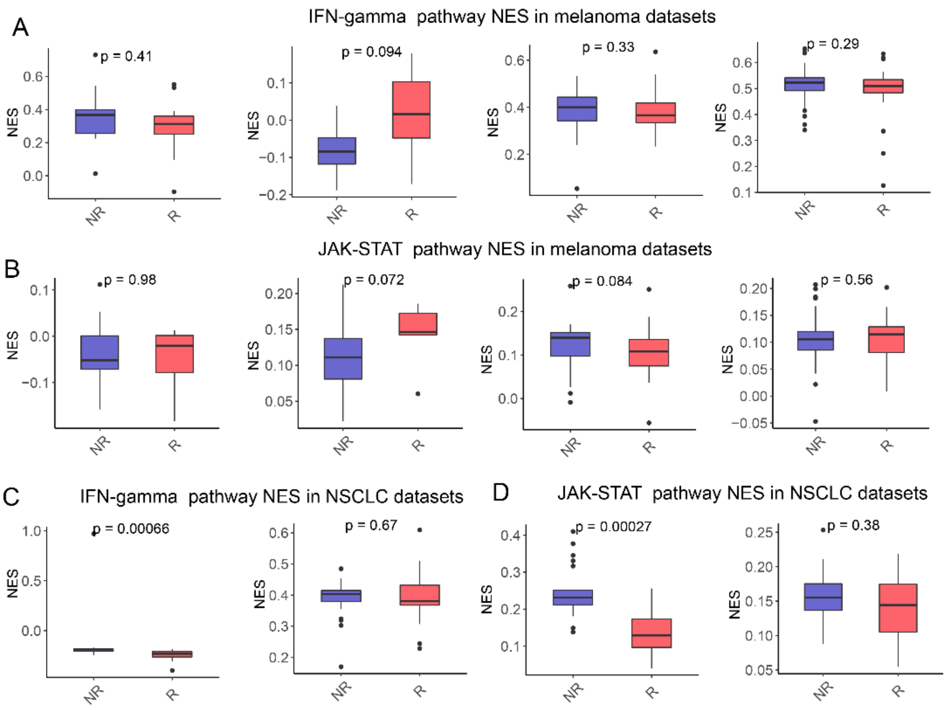

Figure S5. Normalized enrichment score (NES) of mutated genes in IFN gamma and JAK-STAT pathways in samples. (**A**,**B**) NES of mutated genes in IFN gamma (**A**) and JAK-STAT (**B**) pathways in melanoma samples of different datasets. (**C**,**D**) NES of mutated genes in IFN gamma (**C**) and JAK-STAT (**D**) pathways in NSCLC samples of different datasets.

Table S1. Information of samples collected from published data used in this study.

ID	Cancer Type	Sample Location	Stage	Age	Gender	OS (Days)	Status	Anti-Target	Response	Efficacy
SRR3083837	metastatic melanoma	metastasis	IV	62	male	948	Alive	anti-PD1	R	PR
SRR3083839	metastatic melanoma	metastasis	IV	55	male	927	Alive	anti-PD1	R	PR
SRR3083841	metastatic melanoma	metastasis	IV	58	male	691	Dead	anti-PD1	R	PR
SRR3083843	metastatic melanoma	metastasis	IV	71	male	1336	Alive	anti-PD1	R	CR
SRR3083845	metastatic melanoma	metastasis	IV	70	male	980	Dead	anti-PD1	R	PR
SRR3083847	metastatic melanoma	metastasis	IV	45	male	1060	Alive	anti-PD1	R	PR
SRR3083849	metastatic melanoma	metastasis	IV	68	male	1054	Alive	anti-PD1	R	CR
SRR3083851	metastatic melanoma	metastasis	IV	82	male	439	Dead	anti-PD1	R	PR
SRR3083853	metastatic melanoma	metastasis	IV	70	male	974	Dead	anti-PD1	R	PR
SRR3083855	metastatic melanoma	metastasis	IV	53	female	917	Alive	anti-PD1	R	CR
SRR3083857	metastatic melanoma	metastasis	IV	61	male	439	Alive	anti-PD1	R	PR
SRR3083859	metastatic melanoma	metastasis	IV	64	male	1081	Alive	anti-PD1	R	PR
SRR3083861	metastatic melanoma	metastasis	IV	60	male	945	Dead	anti-PD1	R	PR
SRR3083863	metastatic melanoma	metastasis	IV	51	male	882	Alive	anti-PD1	R	PR
SRR3083865	metastatic melanoma	metastasis	IV	66	female	607	Dead	anti-PD1	NR	PD
SRR3083866	metastatic melanoma	metastasis	IV	66	female	607	Dead	anti-PD1	NR	PD
SRR3083868	metastatic melanoma	metastasis	IV	27	female	54	Alive	anti-PD1	NR	PD
SRR3083870	metastatic melanoma	metastasis	IV	55	female	662	Dead	anti-PD1	NR	PD
SRR3083872	metastatic melanoma	metastasis	IV	63	female	337	Dead	anti-PD1	NR	PD
SRR3083874	metastatic melanoma	metastasis	IV	63	male	103	Dead	anti-PD1	NR	PD
SRR3083876	metastatic melanoma	metastasis	IV	84	male	269	Dead	anti-PD1	NR	PD
SRR3083878	metastatic melanoma	metastasis	IV	55	male	182	Dead	anti-PD1	NR	PD
SRR3083880	metastatic melanoma	metastasis	IV	74	male	262	Dead	anti-PD1	NR	PD
SRR3083882	metastatic melanoma	metastasis	IV	59	male	327	Dead	anti-PD1	NR	PD

SRR4289715	metastatic melanoma	metastasis (R leg)	IV	69	male	NA	Alive	anti-PD1	R	CR
SRR4289725	metastatic melanoma	metastasis (R upper arm)	IV	83	male	548	Alive	anti-PD1	R	CR
SRR4289726	metastatic melanoma	metastasis (R upper back)	IV	83	male	548	Alive	anti-PD1	R	CR
SRR4289734	metastatic melanoma	metastasis (groin)	IV	80	female	2177	Alive	anti-PD1	R	CR
SRR4289736	metastatic melanoma	metastasis (abdominal wall)	IV	42	male	418	Alive	anti-PD1	R	CR
SRR4289738	metastatic melanoma	metastasis (flank)	IV	65	female	427	Alive	anti-PD1	R	PR
SRR4289742	metastatic melanoma	metastasis (small bowel)	IV	70	female	364	Alive	anti-PD1	R	PR
SRR4289744	metastatic melanoma	metastasis (cervical)	IV	57	female	448	Alive	anti-PD1	R	PR
SRR4289717	metastatic melanoma	metastasis (cervical)	IV	60	male	387	Alive	anti-PD1	NR	PD
SRR4289719	metastatic melanoma	metastasis (L face)	IV	37	male	467	Dead	anti-PD1	NR	PD
SRR4289721	metastatic melanoma	metastasis (lung)	IV	19	male	186	Dead	anti-PD1	NR	PD
SRR4289723	metastatic melanoma	metastasis (cervical)	IV	67	male	953	Dead	anti-PD1	NR	PD
SRR4289728	metastatic melanoma	metastasis (caecum)	IV	80	female	100	Dead	anti-PD1	NR	PD
SRR4289730	metastatic melanoma	metastasis (sigmoid colon)	IV	47	male	704	Alive	anti-PD1	NR	PD
SRR4289732	metastatic melanoma	metastasis (abdominal wall)	IV	47	male	171	Dead	anti-PD1	NR	PD
SRR4289740	metastatic melanoma	metastasis (armpit)	IV	61	female	837	Dead	anti-PD1	NR	PD
SRR3341169	metastatic melanoma	metastasis (groin)	IV	74	male	2081	alive	anti-CTLA4	R	LTB
SRR3341171	metastatic melanoma	metastasis (scalp)	IV	49	female	767	alive	anti-CTLA4	R	LTB
SRR3341173	metastatic melanoma	metastasis (L thigh)	IV	79	female	1351	dead	anti-CTLA4	R	LTB
SRR3341175	metastatic melanoma	metastasis (gluteal)	IV	54	female	1643	alive	anti-CTLA4	R	LTB
SRR3341177	metastatic melanoma	metastasis (lung)	IV	71	male	1570	alive	anti-CTLA4	R	LTB
SRR3341179	metastatic melanoma	metastasis (breast)	IV	70	male	986	alive	anti-CTLA4	R	LTB
SRR3341181	metastatic melanoma	metastasis (groin)	IV	63	male	1971	alive	anti-CTLA4	R	LTB
SRR3341183	metastatic melanoma	metastasis (elbow)	IV	66	female	694	alive	anti-CTLA4	R	LTB
SRR3341185	melanoma	left axillary lymph nodes	IV	81	male	949	dead	anti-CTLA4	R	LTB

SRR3341189	metastatic melanoma	metastasis (coracoid)	IV	65	male	1935	alive	anti-CTLA4	R	LTB
SRR3341191	metastatic melanoma	metastasis (portal lymph node)	IV	70	male	1606	alive	anti-CTLA4	R	LTB
SRR3341199	melanoma	melanoma (small bowel)	IV	33	male	986	alive	anti-CTLA4	R	LTB
SRR3341201	metastatic melanoma	metastasis (Left parietal brain)	IV	36	female	1205	alive	anti-CTLA4	R	LTB
SRR3341203	metastatic melanoma	metastasis (lymph node)	IV	74	male	1059	alive	anti-CTLA4	R	LTB
SRR3341205	metastasis melanoma	metastasis (vetebral)	IV	62	male	2665	alive	anti-CTLA4	R	LTB
SRR3341207	melanoma	not available	IV	52	male	767	alive	anti-CTLA4	R	LTB
SRR3341209	melanoma	not available	IV	55	male	1241	alive	anti-CTLA4	R	LTB
SRR3341267	metastatic melanoma	metastasis	IV	78	male	1533	alive	anti-CTLA4	R	LTB
SRR3341269	melanoma	lymph nodes	IV	44	male	767	alive	anti-CTLA4	R	LTB
SRR3341271	metastatic melanoma	metastasis	IV	75	male	2227	alive	anti-CTLA4	R	LTB
SRR3341273	metastatic melanoma	metastasis	IV	65	female	2190	alive	anti-CTLA4	R	LTB
SRR3341275	melanoma	lymph nodes	IV	57	female	2227	alive	anti-CTLA4	R	LTB
SRR3341277	metastatic melanoma	metastasis (axillary soft tissue)	IV	43	female	1059	dead	anti-CTLA4	R	LTB
SRR3341279	metastatic melanoma	metastasis (parietal)	IV	70	male	730	alive	anti-CTLA4	R	LTB
SRR3341283	metastatic melanoma	metastasis (lung)	IV	39	female	1424	alive	anti-CTLA4	R	LTB
SRR3341187	metastatic melanoma	metastasis (adrenal gland)	IV	62	male	1460	alive	anti-CTLA4	NR	MNB
SRR3341193	metastatic melanoma	metastasis (small bowel)	IV	49	male	767	alive	anti-CTLA4	NR	MNB
SRR3341195	metastatic melanoma	metastasis (spleen)	IV	71	male	621	alive	anti-CTLA4	NR	MNB
SRR3341197	metastatic melanoma	metastasis (inguinal)	IV	40	female	2628	alive	anti-CTLA4	NR	MNB
SRR3341211	melanoma	L axillary lymph node	IV	68	male	1679	alive	anti-CTLA4	NR	MNB
SRR3341213	metastatic melanoma	metastasis	IV	71	female	986	dead	anti-CTLA4	NR	MNB
SRR3341215	metastatic melanoma	metastasis	IV	43	male	949	dead	anti-CTLA4	NR	MNB
SRR3341217	metastatic melanoma	metastasis (lymph node)	IV	82	female	1533	alive	anti-CTLA4	NR	MNB
SRR3341219	melanoma	groin lymph nodes	IV	68	male	548	dead	anti-CTLA4	NR	MNB
SRR3341221	melanoma	R occipital brain	IV	18	male	292	dead	anti-CTLA4	NR	MNB

SRR3341223	metastatic melanoma	metastasis (gluteal)	IV	68	female	402	dead	anti-CTLA4	NR	MNB
SRR3341225	melanoma	inguinal lymph nodes	IV	50	male	183	dead	anti-CTLA4	NR	MNB
SRR3341227	metastatic melanoma	metastatic	IV	55	male	110	dead	anti-CTLA4	NR	MNB
SRR3341229	metastatic melanoma	metastasis (R elbow)	IV	42	male	621	dead	anti-CTLA4	NR	MNB
SRR3341231	melanoma	not available	IV	54	male	73	dead	anti-CTLA4	NR	MNB
SRR3341233	metastatic melanoma	metastasis	IV	59	male	183	dead	anti-CTLA4	NR	MNB
SRR3341235	metastatic melanoma	metastasis (small bowel)	IV	48	female	146	dead	anti-CTLA4	NR	MNB
SRR3341237	metastatic melanoma	metastasis	IV	79	female	256	dead	anti-CTLA4	NR	MNB
SRR3341239	melanoma	axillary lymph nodes	IV	58	female	183	dead	anti-CTLA4	NR	MNB
SRR3341241	metastatic melanoma	metastasis (L temporal brain)	IV	46	female	438	dead	anti-CTLA4	NR	MNB
SRR3341243	metastatic melanoma	metastasis (R chest wall)	IV	64	male	292	dead	anti-CTLA4	NR	MNB
SRR3341245	metastatic melanoma	metastasis (R axillary)	IV	64	male	292	dead	anti-CTLA4	NR	MNB
SRR3341247	melanoma	axillary lymph nodes	IV	69	male	146	dead	anti-CTLA4	NR	MNB
SRR3341249	metastatic melanoma	metastasis (anterior chest)	IV	67	female	438	dead	anti-CTLA4	NR	MNB
SRR3341251	melanoma	not available	IV	77	male	365	dead	anti-CTLA4	NR	MNB
SRR3341253	melanoma	abdomen	IV	50	male	73	dead	anti-CTLA4	NR	MNB
SRR3341255	melanoma	small bowel resection	IV	64	female	219	dead	anti-CTLA4	NR	MNB
SRR3341257	melanoma	L arm melanoma	IV	74	male	986	dead	anti-CTLA4	NR	MNB
SRR3341259	metastatic melanoma	metastasis (R chest wall)	IV	63	female	438	dead	anti-CTLA4	NR	MNB
SRR3341261	melanoma	left inguinal lymph nodes	IV	38	female	767	dead	anti-CTLA4	NR	MNB
SRR3341263	metastatic melanoma	metastasis (forearm)	IV	90	male	2884	dead	anti-CTLA4	NR	MNB
SRR3341281	metastatic melanoma	metastases (groin lymph node)	IV	61	female	292	dead	anti-CTLA4	NR	MNB
SRR3341285	metastatic melanoma	metastasis (upper back)	IV	55	male	438	dead	anti-CTLA4	NR	MNB
SRR3341289	melanoma	abdominal tumor nodules	IV	52	male	584	dead	anti-CTLA4	NR	MNB
SRR3341293	metastatic melanoma	metastasis (small bowel)	IV	63	female	256	dead	anti-CTLA4	NR	MNB
SRR3341295	metastatic melanoma	metastasis (breast)	IV	50	female	730	dead	anti-CTLA4	NR	MNB
SRR2588053	metastatic melanoma	metastasis	IV	59	male	1005	alive	anti-CTLA4	R	PR

SRR2589393	metastatic melanoma	metastasis	IV	41	male	1047	alive	anti-CTLA4	R	PR
SRR2589478	metastatic melanoma	metastasis	IV	70	male	1048	dead	anti-CTLA4	R	PR
SRR2661602	metastatic melanoma	metastasis	IV	45	male	1539	alive	anti-CTLA4	R	PR
SRR2661932	metastatic melanoma	metastasis	IV	68	male	301	dead	anti-CTLA4	R	PR
SRR2663954	metastatic melanoma	metastasis	IV	74	female	971	alive	anti-CTLA4	R	PR
SRR2674312	metastatic melanoma	metastasis	IV	81	male	676	alive	anti-CTLA4	R	PR
SRR2677533	metastatic melanoma	metastasis	IV	73	male	914	alive	anti-CTLA4	R	PR
SRR2679057	metastatic melanoma	metastasis	IV	50	female	853	dead	anti-CTLA4	NR	SD
SRR2681131	metastatic melanoma	metastasis	IV	61	male	1379	alive	anti-CTLA4	NR	PD
SRR2693653	metastatic melanoma	metastasis	IV	44	female	653	alive	anti-CTLA4	NR	SD
SRR2702801	metastatic melanoma	metastasis	IV	81	male	676	dead	anti-CTLA4	NR	SD
SRR2703695	metastatic melanoma	metastasis	IV	22	male	1027	alive	anti-CTLA4	NR	PD
SRR2717684	metastatic melanoma	metastasis	IV	68	female	811	dead	anti-CTLA4	NR	PD
SRR2723814	metastatic melanoma	metastasis	IV	71	male	987	alive	anti-CTLA4	R	PR
SRR2727854	metastatic melanoma	metastasis	IV	61	male	808	alive	anti-CTLA4	NR	PD
SRR2740217	metastatic melanoma	metastasis	IV	54	male	749	alive	anti-CTLA4	NR	PD
SRR2757072	metastatic melanoma	metastasis	IV	69	male	801	dead	anti-CTLA4	R	PR
SRR2757601	metastatic melanoma	metastasis	IV	67	male	1487	alive	anti-CTLA4	R	CR
SRR2765066	metastatic melanoma	metastasis	IV	48	male	724	dead	anti-CTLA4	NR	SD
SRR2765304	metastatic melanoma	metastasis	IV	60	female	989	alive	anti-CTLA4	NR	SD
SRR2770259	metastatic melanoma	metastasis	IV	78	male	732	dead	anti-CTLA4	NR	PD
SRR2770409	metastatic melanoma	metastasis	IV	65	male	1039	alive	anti-CTLA4	R	CR
SRR2771788	metastatic melanoma	metastasis	IV	77	male	1184	dead	anti-CTLA4	NR	PD
SRR2772131	metastatic melanoma	metastasis	IV	39	female	1478	dead	anti-CTLA4	NR	SD
SRR2772336	metastatic melanoma	metastasis	IV	77	male	641	alive	anti-CTLA4	R	PR

SRR2772697	metastatic melanoma	metastasis	IV	71	male	442	dead	anti-CTLA4	R	PR
SRR2772803	metastatic melanoma	metastasis	IV	21	male	1029	alive	anti-CTLA4	R	PR
SRR2772900	metastatic melanoma	metastasis	IV	78	male	1106	alive	anti-CTLA4	R	CR
SRR2773166	metastatic melanoma	metastasis	IV	42	female	1632	alive	anti-CTLA4	NR	SD
SRR2773334	metastatic melanoma	metastasis	IV	67	male	790	alive	anti-CTLA4	NR	PD
SRR2773533	metastatic melanoma	metastasis	IV	82	male	1326	alive	anti-CTLA4	R	R
SRR2777240	metastatic melanoma	metastasis	IV	51	male	842	alive	anti-CTLA4	NR	PD
SRR2777655	metastatic melanoma	metastasis	IV	36	male	1034	alive	anti-CTLA4	NR	SD
SRR2779374	metastatic melanoma	metastasis	IV	36	male	1050	alive	anti-CTLA4	NR	SD
SRR2780332	metastatic melanoma	metastasis	IV	55	female	798	alive	anti-CTLA4	NR	PD
SRR2891925	metastatic melanoma	metastasis	IV	57	female	688	alive	anti-CTLA4	R	PR
SRR2587585	metastatic melanoma	metastasis	IV	55	male	293	dead	anti-CTLA4	NR	SD
SRR2587785	metastatic melanoma	metastasis	IV	61	female	100	dead	anti-CTLA4	NR	PD
SRR2588166	metastatic melanoma	metastasis	IV	57	female	271	dead	anti-CTLA4	NR	PD
SRR2588422	metastatic melanoma	metastasis	IV	57	female	140	dead	anti-CTLA4	NR	PD
SRR2588549	metastatic melanoma	metastasis	IV	46	male	122	dead	anti-CTLA4	NR	PD
SRR2589158	metastatic melanoma	metastasis	IV	69	male	325	dead	anti-CTLA4	NR	PD
SRR2589276	metastatic melanoma	metastasis	IV	71	female	38	dead	anti-CTLA4	NR	PD
SRR2589694	metastatic melanoma	metastasis	IV	50	female	115	dead	anti-CTLA4	NR	PD
SRR2589808	metastatic melanoma	metastasis	IV	83	male	106	dead	anti-CTLA4	NR	PD
SRR2663198	metastatic melanoma	metastasis	IV	74	male	34	dead	anti-CTLA4	NR	PD
SRR2665040	metastatic melanoma	metastasis	IV	31	male	77	dead	anti-CTLA4	NR	NR
SRR2668212	metastatic melanoma	metastasis	IV	74	male	139	dead	anti-CTLA4	NR	PD
SRR2670626	metastatic melanoma	metastasis	IV	76	male	542	dead	anti-CTLA4	NR	PD
SRR2670951	metastatic melanoma	metastasis	IV	63	male	137	dead	anti-CTLA4	NR	PD

SRR2675035	metastatic melanoma	metastasis	IV	33	male	161	dead	anti-CTLA4	NR	PD
SRR2675269	metastatic melanoma	metastasis	IV	45	male	145	dead	anti-CTLA4	NR	PD
SRR2677161	metastatic melanoma	metastasis	IV	68	male	89	dead	anti-CTLA4	NR	PD
SRR2677297	metastatic melanoma	metastasis	IV	65	male	211	dead	anti-CTLA4	NR	PD
SRR2678485	metastatic melanoma	metastasis	IV	76	male	602	dead	anti-CTLA4	NR	PD
SRR2679013	metastatic melanoma	metastasis	IV	36	female	160	dead	anti-CTLA4	NR	PD
SRR2679085	metastatic melanoma	metastasis	IV	48	male	108	dead	anti-CTLA4	NR	PD
SRR2680970	metastatic melanoma	metastasis	IV	73	male	193	dead	anti-CTLA4	NR	PD
SRR2682345	metastatic melanoma	metastasis	IV	54	female	104	dead	anti-CTLA4	NR	PD
SRR2684746	metastatic melanoma	metastasis	IV	58	male	99	dead	anti-CTLA4	NR	PD
SRR2689326	metastatic melanoma	metastasis	IV	72	male	147	dead	anti-CTLA4	NR	PD
SRR2692147	metastatic melanoma	metastasis	IV	55	female	211	dead	anti-CTLA4	NR	PD
SRR2706574	metastatic melanoma	metastasis	IV	69	female	86	dead	anti-CTLA4	NR	NR
SRR2707236	metastatic melanoma	metastasis	IV	54	female	628	dead	anti-CTLA4	NR	PD
SRR2709858	metastatic melanoma	metastasis	IV	29	male	113	dead	anti-CTLA4	NR	PD
SRR2712344	metastatic melanoma	metastasis	IV	71	male	153	dead	anti-CTLA4	NR	PD
SRR2726249	metastatic melanoma	metastasis	IV	73	male	208	dead	anti-CTLA4	NR	PD
SRR2731893	metastatic melanoma	metastasis	IV	29	female	81	dead	anti-CTLA4	NR	PD
SRR2732125	metastatic melanoma	metastasis	IV	43	female	250	dead	anti-CTLA4	NR	PD
SRR2741440	metastatic melanoma	metastasis	IV	70	male	360	dead	anti-CTLA4	NR	PD
SRR2747280	metastatic melanoma	metastasis	IV	66	male	466	dead	anti-CTLA4	NR	PD
SRR2747991	metastatic melanoma	metastasis	IV	78	male	146	dead	anti-CTLA4	NR	PD
SRR2753222	metastatic melanoma	metastasis	IV	45	female	237	dead	anti-CTLA4	NR	SD
SRR2753967	metastatic melanoma	metastasis	IV	35	female	84	dead	anti-CTLA4	NR	PD
SRR2760983	metastatic melanoma	metastasis	IV	75	male	288	dead	anti-CTLA4	NR	PD

SRR2762331	metastatic melanoma	metastasis	IV	69	male	83	dead	anti-CTLA4	NR	PD
SRR2767414	metastatic melanoma	metastasis	IV	50	male	408	dead	anti-CTLA4	NR	PD
SRR2767760	metastatic melanoma	metastasis	IV	59	female	652	dead	anti-CTLA4	NR	PD
SRR2768297	metastatic melanoma	metastasis	IV	18	male	539	alive	anti-CTLA4	NR	PD
SRR2768790	metastatic melanoma	metastasis	IV	32	male	50	dead	anti-CTLA4	NR	PD
SRR2769286	metastatic melanoma	metastasis	IV	38	male	532	dead	anti-CTLA4	NR	PD
SRR2770067	metastatic melanoma	metastasis	IV	44	male	205	dead	anti-CTLA4	NR	PD
SRR2770550	metastatic melanoma	metastasis	IV	67	female	90	dead	anti-CTLA4	NR	NR
SRR2771331	metastatic melanoma	metastasis	IV	52	female	54	dead	anti-CTLA4	NR	PD
SRR2771975	metastatic melanoma	metastasis	IV	39	male	79	dead	anti-CTLA4	NR	PD
SRR2772054	metastatic melanoma	metastasis	IV	25	female	332	dead	anti-CTLA4	NR	PD
SRR2773626	metastatic melanoma	metastasis	IV	47	female	70	dead	anti-CTLA4	NR	PD
SRR2773750	metastatic melanoma	metastasis	IV	73	male	140	dead	anti-CTLA4	NR	PD
SRR2774183	metastatic melanoma	metastasis	IV	77	male	65	dead	anti-CTLA4	NR	PD
SRR2774324	metastatic melanoma	metastasis	IV	68	male	235	dead	anti-CTLA4	NR	PD
SRR2774418	metastatic melanoma	metastasis	IV	48	female	163	dead	anti-CTLA4	NR	PD
SRR2774559	metastatic melanoma	metastasis	IV	76	male	320	dead	anti-CTLA4	NA	PD
SRR2774969	metastatic melanoma	metastasis	IV	63	male	224	dead	anti-CTLA4	NR	PD
SRR2775168	metastatic melanoma	metastasis	IV	49	male	123	dead	anti-CTLA4	NR	PD
SRR2775456	metastatic melanoma	metastasis	IV	68	male	204	dead	anti-CTLA4	NR	PD
SRR2775592	metastatic melanoma	metastasis	IV	64	male	128	dead	anti-CTLA4	NR	PD
SRR2776179	metastatic melanoma	metastasis	IV	86	male	272	dead	anti-CTLA4	NR	PD
SRR2776798	metastatic melanoma	metastasis	IV	75	female	37	dead	anti-CTLA4	NR	PD
SRR2777326	metastatic melanoma	metastasis	IV	83	male	458	dead	anti-CTLA4	NR	PD
SRR2777738	metastatic melanoma	metastasis	IV	43	female	313	dead	anti-CTLA4	NR	PD

SRR2777872	metastatic melanoma	metastasis	IV	71	female	193	dead	anti-CTLA4	NR	PD
SRR2777977	metastatic melanoma	metastasis	IV	62	male	492	dead	anti-CTLA4	NR	PD
SRR2778175	metastatic melanoma	metastasis	IV	79	male	152	dead	anti-CTLA4	NR	PD
SRR2778237	metastatic melanoma	metastasis	IV	69	male	250	dead	anti-CTLA4	NR	PD
SRR2778365	metastatic melanoma	metastasis	IV	76	male	45	dead	anti-CTLA4	NR	PD
SRR2778705	metastatic melanoma	metastasis	IV	68	male	67	dead	anti-CTLA4	NR	NR
SRR2779219	metastatic melanoma	metastasis	IV	36	male	224	dead	anti-CTLA4	NR	PD
SRR2779901	metastatic melanoma	metastasis	IV	59	male	175	dead	anti-CTLA4	NR	PD
SRR2780177	metastatic melanoma	metastasis	IV	32	male	257	dead	anti-CTLA4	NR	SD
SRR2648109	NSCLC	Lung	IV	57	male	435	NA	anti-PD1	R	PR
SRR2648113	NSCLC	Lung	IV	61	female	294	NA	anti-PD1	R	PR
SRR2648117	NSCLC	Lung	IV	65	female	126	NA	anti-PD1	R	PR
SRR2648119	NSCLC	Lung	IV	80	male	249	NA	anti-PD1	R	PR
SRR2648123	NSCLC	Lung	IV	63	female	440	NA	anti-PD1	R	PR
SRR2648133	NSCLC	Lung	IV	66	male	438	NA	anti-PD1	R	PR
SRR2648141	NSCLC	Lung	IV	78	female	313	NA	anti-PD1	R	PR
SRR2648143	NSCLC	Lung	IV	60	female	498	NA	anti-PD1	R	PR
SRR2648147	NSCLC	Lung	IV	63	female	251	NA	anti-PD1	R	PR
SRR2648149	NSCLC	Lung	IV	68	male	819	NA	anti-PD1	R	PR
SRR2648151	NSCLC	Lung	IV	64	female	377	NA	anti-PD1	R	PR
SRR2648169	NSCLC	Lung	IV	57	female	133	NA	anti-PD1	R	PR
SRR2648103	NSCLC	Lung	IV	59	male	251	NA	anti-PD1	NR	SD
SRR2648105	NSCLC	Lung	IV	64	male	55	NA	anti-PD1	NR	PD
SRR2648107	NSCLC	Lung	IV	73	female	195	NA	anti-PD1	NR	SD
SRR2648111	NSCLC	Lung	IV	57	male	119	NA	anti-PD1	NR	SD
SRR2648115	NSCLC	Lung	IV	50	male	57	NA	anti-PD1	NR	PD
SRR2648121	NSCLC	Lung	IV	59	male	56	NA	anti-PD1	NR	PD
SRR2648125	NSCLC	Lung	IV	60	male	98	NA	anti-PD1	NR	PD
SRR2648127	NSCLC	Lung	IV	64	female	244	NA	anti-PD1	NR	SD
SRR2648129	NSCLC	Lung	IV	62	female	105	NA	anti-PD1	NR	SD
SRR2648131	NSCLC	Lung	IV	56	female	189	NA	anti-PD1	NR	SD
SRR2648135	NSCLC	Lung	IV	56	male	54	NA	anti-PD1	NR	PD
SRR2648137	NSCLC	Lung	IV	41	female	56	NA	anti-PD1	NR	PD
SRR2648139	NSCLC	Lung	IV	63	male	41	NA	anti-PD1	NR	PD
SRR2648145	NSCLC	Lung	IV	71	male	64	NA	anti-PD1	NR	PD
SRR2648153	NSCLC	Lung	IV	59	male	250	NA	anti-PD1	NR	SD
SRR2648155	NSCLC	Lung	IV	51	female	103	NA	anti-PD1	NR	PD
SRR2648157	NSCLC	Lung	IV	66	male	62	NA	anti-PD1	NR	PD
SRR2648159	NSCLC	Lung	IV	71	female	124	NA	anti-PD1	NR	SD
SRR2648161	NSCLC	Lung	IV	57	female	188	NA	anti-PD1	NR	SD

SRR2648163	NSCLC	Lung	IV	49	male	57	NA	anti-PD1	NR	PD
SRR2648165	NSCLC	Lung	IV	68	female	250	NA	anti-PD1	NR	SD
SRR2648167	NSCLC	Lung	IV	76	female	58	NA	anti-PD1	NR	PD
19_indel.vcf.gz	NSCLC	Lung	IV	59	male	863	NA	anti-PD1+anti-CTLA4	R	PR
34_indel.vcf.gz	NSCLC	Lung	IV	76	male	670	NA	anti-PD1+anti-CTLA4	R	PR
2_indel.vcf.gz	NSCLC	Lung	IV	72	male	204	NA	anti-PD1+anti-CTLA4	R	PR
7_indel.vcf.gz	NSCLC	Lung	IV	56	male	1158	NA	anti-PD1+anti-CTLA4	R	PR
40_indel.vcf.gz	NSCLC	Lung	IV	73	male	243	NA	anti-PD1+anti-CTLA4	R	PR
30_indel.vcf.gz	NSCLC	Lung	IV	75	male	636	NA	anti-PD1+anti-CTLA4	R	PR
21_indel.vcf.gz	NSCLC	Lung	IV	74	female	648	NA	anti-PD1+anti-CTLA4	R	CR
26_indel.vcf.gz	NSCLC	Lung	IV	60	female	690	NA	anti-PD1+anti-CTLA4	R	PR
41_indel.vcf.gz	NSCLC	Lung	IV	53	male	406	NA	anti-PD1+anti-CTLA4	R	CR
37_indel.vcf.gz	NSCLC	Lung	IV	57	female	571	NA	anti-PD1+anti-CTLA4	R	PR
12_indel.vcf.gz	NSCLC	Lung	IV	82	male	785	NA	anti-PD1+anti-CTLA4	R	PR
32_indel.vcf.gz	NSCLC	Lung	IV	50	female	570	NA	anti-PD1+anti-CTLA4	R	CR
33_indel.vcf.gz	NSCLC	Lung	IV	74	male	649	NA	anti-PD1+anti-CTLA4	R	PR
38_indel.vcf.gz	NSCLC	Lung	IV	66	male	235	NA	anti-PD1+anti-CTLA4	R	PR
23_indel.vcf.gz	NSCLC	Lung	IV	75	female	240	NA	anti-PD1+anti-CTLA4	R	PR

18_indel.vcf.gz	NSCLC	Lung	IV	69	female	892	NA	anti-PD1+anti-CTLA4	R	PR
14_indel.vcf.gz	NSCLC	Lung	IV	43	female	50	NA	anti-PD1+anti-CTLA4	NR	PD
24_indel.vcf.gz	NSCLC	Lung	IV	85	female	68	NA	anti-PD1+anti-CTLA4	NR	PD
29_indel.vcf.gz	NSCLC	Lung	IV	50	female	78	NA	anti-PD1+anti-CTLA4	NR	PD
35_indel.vcf.gz	NSCLC	Lung	IV	66	female	70	NA	anti-PD1+anti-CTLA4	NR	PD
3_indel.vcf.gz	NSCLC	Lung	IV	61	female	44	NA	anti-PD1+anti-CTLA4	NR	SD
10_indel.vcf.gz	NSCLC	Lung	IV	72	male	163	NA	anti-PD1+anti-CTLA4	NR	SD
20_indel.vcf.gz	NSCLC	Lung	IV	54	male	43	NA	anti-PD1+anti-CTLA4	NR	PD
8_indel.vcf.gz	NSCLC	Lung	IV	58	male	90	NA	anti-PD1+anti-CTLA4	NR	SD
15_indel.vcf.gz	NSCLC	Lung	IV	53	female	1	NA	anti-PD1+anti-CTLA4	NR	PD
25_indel.vcf.gz	NSCLC	Lung	IV	65	male	664	NA	anti-PD1+anti-CTLA4	NR	SD
31_indel.vcf.gz	NSCLC	Lung	IV	67	female	70	NA	anti-PD1+anti-CTLA4	NR	PD
36_indel.vcf.gz	NSCLC	Lung	IV	62	female	400	NA	anti-PD1+anti-CTLA4	NR	SD
4_indel.vcf.gz	NSCLC	Lung	IV	64	male	113	NA	anti-PD1+anti-CTLA4	NR	SD
11_indel.vcf.gz	NSCLC	Lung	IV	58	female	235	NA	anti-PD1+anti-CTLA4	NR	SD
9_indel.vcf.gz	NSCLC	Lung	IV	68	female	233	NA	anti-PD1+anti-CTLA4	NR	SD

16_indel.vcf.gz	NSCLC	Lung	IV	67	male	109	NA	anti-PD1+anti-CTLA4	NR	SD
42_indel.vcf.gz	NSCLC	Lung	IV	59	male	118	NA	anti-PD1+anti-CTLA4	NR	SD
5_indel.vcf.gz	NSCLC	Lung	IV	57	male	227	NA	anti-PD1+anti-CTLA4	NR	SD
22_indel.vcf.gz	NSCLC	Lung	IV	72	male	75	NA	anti-PD1+anti-CTLA4	NR	PD
17_indel.vcf.gz	NSCLC	Lung	IV	68	female	363	NA	anti-PD1+anti-CTLA4	NR	SD
27_indel.vcf.gz	NSCLC	Lung	IV	55	male	123	NA	anti-PD1+anti-CTLA4	NR	SD
28_indel.vcf.gz	NSCLC	Lung	IV	77	female	512	NA	anti-PD1+anti-CTLA4	NR	SD
1_indel.vcf.gz	NSCLC	Lung	IV	70	female	42	NA	anti-PD1+anti-CTLA4	NR	PD
43_indel.vcf.gz	NSCLC	Lung	IV	58	female	153	NA	anti-PD1+anti-CTLA4	NR	SD
6_indel.vcf.gz	NSCLC	Lung	IV	67	female	111	NA	anti-PD1+anti-CTLA4	NR	SD
13_indel.vcf.gz	NSCLC	Lung	IV	76	male	719	NA	anti-PD1+anti-CTLA4	NR	SD

Response (R) includes: complete response (CR), partial response (PR) and long-term-benefit (LTB). Non-responders (NR) includes: stable disease (SD), progressive disease (PD), and minimal-or-no-benefit (MNB). OS: overall survival.

Table S2. Genes in biological pathways used for calculating P-TMB.

Neutrophil extracellular trap formation	FCGR3A, FCGR3B, SYK, MAP3K7, RAF1, MAP2K1, MAP2K2, MAPK1, MAPK3, SIGLEC9, CYBB, NCF1, CYBA, NCF2, NCF4, RAC1, RAC2, TLR7, TLR8, ELANE, MPO, ACTG1, ACTB, VDAC1, VDAC2, VDAC3, SLC25A4, SLC25A5, SLC25A6, SLC25A31, PPIF, PADI4, IGH, FCGR1A, FCGR2A, ITGAM, ITGB2, ITGAL, CLEC7A, SRC, PLCB1, PLCB2, PLCB3, PLCB4, PLCG1, PLCG2, PRKCA, PRKCB, PRKCG, ATG7, FPR1, FPR3, FPR2, PIK3CA, PIK3CD, PIK3CB, PIK3R1, PIK3R2, PIK3R3, AKT1, AKT2, AKT3, MTOR, NFKB1, RELA, C3, CR1, CR1L, C5, C5AR1, HMGB1, TLR2, TLR4, MAPK11, MAPK12, MAPK13, MAPK14, ITGA2B, ITGB3, FGA, FGB, FGG, GP1BA, VWF, SELP, SELPLG, AGER, CASP4, CASP1, GSDMD, HAT1, H2AX, H2AC20, H2AC12, H2AC1, H2AW, H2AB3, H2AC8, H2AC4, MACROH2A2, MACROH2A1, H2AC19, H2AJ, H2AB1, H2AC17, H2AC18, H2AC11, H2AC21, H2AZ2, H2AC7, H2AZ1, H2AC15, H2AC6, H2AC13, H2AC14, H2AC16, H2AB2, H2BC15, H2BC9, H2BC14, H2BW1, H2BC1, H2BC18, H2BC17, H2BC3, H2BC8, H2BC13, H2BC4, H2BC5, H2BC6, H2BC10, H2BC21, H2BC11, H2BC12, H2BU1, H2BC7, H2BW2

MAPK signaling pathway	AKT1, AKT2, AKT3, ARRB1, ARRB2, ATF2, ATF4, BDNF, BRAF, CACNA1A, CACNA1B, CACNA1C, CACNA1D, CACNA1E, CACNA1F, CACNA1G, CACNA1H, CACNA1I, CACNA1S, CACNA2D1, CACNA2D2, CACNA2D3, CACNA2D4, CACNB1, CACNB2, CACNB3, CACNB4, CACNG1, CACNG2, CACNG3, CACNG4, CACNG5, CACNG6, CACNG7, CACNG8, CASP3, CD14, CDC25B, CDC42, CHP1, CHP2, CHUK, CRK, CRKL, DAXX, DDIT3, DUSP1, DUSP10, DUSP14, DUSP16, DUSP2, DUSP3, DUSP4, DUSP5, DUSP6, DUSP7, DUSP8, DUSP9, ECSIT, EGF, EGFR, ELK1, ELK4, FAS, FASLG, FGF1, FGF10, FGF11, FGF12, FGF13, FGF14, FGF16, FGF17, FGF18, FGF19, FGF2, FGF20, FGF21, FGF22, FGF23, FGF3, FGF4, FGF5, FGF6, FGF7, FGF8, FGF9, FGFR1, FGFR2, FGFR3, FGFR4, FLNA, FLNB, FLNC, FOS, GADD45A, GADD45B, GADD45G, GNA12, GNG12, GRB2, HRAS, HSPA1A, HSPA1B, HSPA1L, HSPA2, HSPA6, HSPA8, HSPB1, IKBKB, IKBKG, IL1A, IL1B, IL1R1, IL1R2, JMJD7-PLA2G4B, JUN, JUND, KRAS, LAMTOR3, MAP2K1, MAP2K2, MAP2K3, MAP2K4, MAP2K5, MAP2K6, MAP2K7, MAP3K1, MAP3K11, MAP3K12, MAP3K13, MAP3K14, MAP3K2, MAP3K20, MAP3K3, MAP3K4, MAP3K5, MAP3K6, MAP3K7, MAP3K8, MAP4K1, MAP4K2, MAP4K3, MAP4K4, MAPK1, MAPK10, MAPK11, MAPK12, MAPK13, MAPK14, MAPK3, MAPK7, MAPK8, MAPK8IP1, MAPK8IP2, MAPK8IP3, MAPK9, MAPKAPK2, MAPKAPK3, MAPKAPK5, MAPT, MAX, MECOM, MEF2C, MKNK1, MKNK2, MOS, MRAS, MYC, NF1, NFATC2, NFATC4, NFKB1, NFKB2, NGF, NLK, NR4A1, NRAS, NTF3, NTF4, NTRK1, NTRK2, PAK1, PAK2, PDGFA, PDGFB, PDGFRA, PDGFRB, PLA2G10, PLA2G12A, PLA2G12B, PLA2G1B, PLA2G2A, PLA2G2C, PLA2G2D, PLA2G2E, PLA2G2F, PLA2G3, PLA2G4A, PLA2G4B, PLA2G4E, PLA2G5, PLA2G6, PPM1A, PPM1B, PPP3CA, PPP3CB, PPP3CC, PPP3R1, PPP3R2, PPP5C, PRKACA, PRKACB, PRKACG, PRKCA, PRKCB, PRKCG, PRKX, PTPN5, PTPN7, PTPRR, RAC1, RAC2, RAC3, RAF1, RAP1A, RAP1B, RAPGEF2, RASA1, RASA2, RASGRF1, RASGRF2, RASGRP1, RASGRP2, RASGRP3, RASGRP4, RELA, RELB, RPS6KA1, RPS6KA2, RPS6KA3, RPS6KA4, RPS6KA5, RPS6KA6, RRAS, RRAS2, SOS1, SOS2, SRF, STK3, STK4, STMN1, TAB1, TAB2, TAOK1, TAOK2, TAOK3, TGFB1, TGFB2, TGFB3, TGFBR1, TGFBR2, TNF, TNFRSF1A, TP53, TRAF2, TRAF6
Base excision repair	APEX1, APEX2, FEN1, HMGB1, HMGB1P1, HMGB1P40, LIG1, LIG3, MBD4, MPG, MUTYH, NEIL1, NEIL2, NEIL3, NTHL1, OGG1, PARP1, PARP2, PARP3, PARP4, PCNA, POLB, POLD1, POLD2, POLD3, POLD4, POLE, POLE2, POLE3, POLE4, POLL, SMUG1, TDG, UNG, XRCC1
Mismatch repair	SSBP1, PMS2, MLH1, MSH6, MSH2, MSH3, MLH3, RFC1, RFC4, RFC2, RFC5, RFC3, PCNA, EXO1, RPA1, RPA2, RPA3, RPA4, POLD1, POLD2, POLD3, POLD4, LIG1
Chemokine Receptors	C5AR1, ACKR2, CCR1, CCR10, CCR3, CCR4, CCR5, CCR6, CCR7, CCR8, CCR9, ACKR4, CCRL2, CMKLR1, CX3CR1, CXCR3, CXCR4, CXCR5, CXCR6, ACKR3, CYSLTR1, CYSLTR2, ACKR1, EDNRA, EDNRB, FPR1, FPR2, GPR17, GPR32, GPR33, PTGDR2, C5AR2, CXCR1, CXCR2, LTB4R, LTB4R2, PLAUR, PLXNA1, PLXNA2, PLXNA3, PLXNA4, PLXNB1, PLXNB2, PLXNB3, PLXNC1, PLXND1, PTAFR, ROBO1, ROBO2, ROBO3, RXFP3, XCR1
Autoimmune thyroid disease	CD28, CD40, CD40LG, CD80, CD86, CGA, CTLA4, FAS, FASLG, GZMB, HLA-A, HLA-B, HLA-C, HLA-DMA, HLA-DMB, HLA-DOA, HLA-DOB, HLA-DPA1, HLA-DPB1, HLA-DQA1, HLA-DQA2, HLA-DQB1, HLA-DRA, HLA-DRB1, HLA-DRB3, HLA-DRB4, HLA-DRB5, HLA-E, HLA-F, HLA-G, IFNA1, IFNA10, IFNA13, IFNA14, IFNA16, IFNA17, IFNA2, IFNA21, IFNA4, IFNA5, IFNA6, IFNA7, IFNA8, IL10, IL2, IL4, IL5, PRF1, TG, TPO, TSHB, TSHR
Pentose phosphate pathway	ALDOA, ALDOB, ALDOC, DERA, FBP1, FBP2, G6PD, GPI, H6PD, PFKL, PFKM, PFKP, PGD, PGLS, PGM1, PGM2, PRPS1, PRPS1L1, PRPS2, RBKS, RPE, RPEL1, RPIA, TALDO1, TKT, TKTL1, TKTL2
Regulation of autophagy	ATG12, ATG3, ATG4A, ATG4B, ATG4C, ATG4D, ATG5, ATG7, BECN1, BECN2, GABARAP, GABARAPL1, GABARAPL2, IFNA1, IFNA10, IFNA13, IFNA14, IFNA16, IFNA17, IFNA2, IFNA21, IFNA4, IFNA5, IFNA6, IFNA7, IFNA8, IFNG, INS, PIK3C3, PIK3R4, PRKAA1, PRKAA2, ULK1, ULK2, ULK3
Citrate cycle (TCA cycle)	ACLY, ACO1, ACO2, CS, DLAT, DLD, DLST, FH, IDH1, IDH2, IDH3A, IDH3B, IDH3G, MDH1, MDH2, OGDH, OGDHL, PC, PCK1, PCK2, PDHA1, PDHA2, PDHB, SDHA, SDHB, SDHC, SDHD, SUCLA2, SUCLG1, SUCLG2, SUCLG2P2

Terpenoid backbone biosynthesis	ACAT1, ACAT2, DHDDS, FDPS, GGPS1, HMGCR, HMGCS1, HMGCS2, IDI1, IDI2, MVD, MVK, PDSS1, PDSS2, PMVK
DNA checkpoints	DNACHK, MDC1, ATM, ATR, BRCA1, CHEK1, CHEK2, TP53BP1
TGFb Family Members Receptors	ACVR1B, ACVR1C, ACVR2A, ACVR2B, ACVRL1, AMHR2, BMPR1A, BMPR1B, BMPR2, TGFBR1, TGFBR2, TGFBR3
